# The Abdomen in “Thoracoabdominal” Cannot Be Ignored: Abdominal Compartment Syndrome Complicating Extracorporeal Life Support

**DOI:** 10.1155/2014/351340

**Published:** 2014-05-08

**Authors:** Arthur J. Lee, Bryan J. Wells, Rosaleen Chun, Chad G. Ball, Andrew. W. Kirkpatrick

**Affiliations:** ^1^University of Calgary, Calgary, AB, Canada; ^2^Departments of Surgery, Foothills Medical Centre, 1403 29 Street NW, Calgary, AB, Canada T2N 2T9; ^3^Critical Care Medicine, Foothills Medical Centre, 1403 29 Street NW, Calgary, AB, Canada T2N 2T9; ^4^The Regional Trauma Program, Foothills Medical Centre, 1403 29 Street NW, Calgary, AB, Canada T2N 2T9; ^5^Anesthesia, Foothills Medical Centre, 1403 29 St NW, Calgary, Alberta, Canada T2N 2T9

## Abstract

Extracorporeal life support (ECLS) is an incredible life-saving measure that is being used ever more frequently in the care of the critically ill. Management of these patients requires extreme vigilance on the part of the care providers in recognizing and addressing the complications and challenges that may arise. We present a case of overt abdominal compartment syndrome (ACS) in a previously well young male on ECLS with a history of trauma, submersion, hypothermia, and no intra-abdominal injuries. The patient developed ACS soon after ECLS was initiated which resulted in drastically compromised flow rates. Taking into account the patient's critical status, an emergent laparotomy was performed in the intensive care unit which successfully resolved the ACS and restored ECLS flow. The patient had an unremarkable course following and was weaned off ECLS but unfortunately died from his original anoxic injury. This case highlights several salient points: first, care of patients on ECLS is challenging and multiple etiologies can affect our ability to manage these patients; second, intra-abdominal pressures should be monitored liberally in the critically ill, especially in patients on ECLS; third, protocols for emergent operative treatment outside of traditional operating rooms should be established and care providers should be prepared for these situations.

## 1. Introduction

Extracorporeal life support (ECLS) is being utilized ever more frequently for an increasing array of critical care indications. In our experience, patients are typically unstable and catastrophically ill prior to the initiation of ECLS. In addition to the obvious distress amongst patients themselves, these scenarios are also stressful for the medical staff who fear for the patient's demise. Thus, once ECLS is initiated it is only “human” that care providers will relax and assume that the hemodynamics will now be “stabilized” through the application of this extraordinary life support technology.

Vigilance at the bedside is always required of those caring for the critically ill. This is especially true in relation to intra-abdominal hypertension (IAH) which appears to be a nearly ubiquitous complication of critical illness [1], yet it is one that is often ignored [2]. Recently, it has been recognized that increased intra-abdominal pressure (IAP) can influence the physiology of both adjacent and anatomically distant compartments in a syndrome known as polycompartment syndrome [3, 4].

We present a case of overt abdominal compartment syndrome (ACS) in a young male with acute respiratory distress syndrome (ARDS) on ECLS. The patient had no abdominal injuries and yet developed ACS, significantly compromising the ECLS function. Emergent treatment with bedside laparotomy successfully restored ECLS flows.

## 2. Case Summary

A previously healthy 23-year-old male was involved in a single vehicle rollover into standing water in freezing conditions. He was found unresponsive and submerged and had periods of asystole during prehospital transfer. He was resuscitated despite a Glasgow Coma Scale of 3_T_ and dilated fixed pupils upon arrival with a temperature of 31.6°C. He demonstrated profound acidemia (pH 6.80), hypercapnia (pCO_2_ 94), and hyperprolactinemia (13.0 mmol/L). Imaging with pan-computed tomography (CT) revealed bilateral pulmonary infiltrates and a small right-sided pneumothorax. Other injuries included fractures of the 2nd and 3rd cervical vertebrae with grade 2 anterolisthesis and a closed right midshaft radius fracture. Of note, no intra-abdominal injuries were appreciated on trauma-protocolized imaging of the abdomen. The physical examination was otherwise unremarkable.

Bilateral tube thoracostomy and pressure controlled mechanical ventilation did not improve ventilation. Repeat arterial blood gases continued to reveal profound gas-exchange abnormalities consistent with severe acute respiratory distress syndrome (ARDS) (PaO_2_/FiO_2_ 52) [5]. High frequency oscillation ventilation was therefore initiated without significant improvement and oxygen saturations remained between 70 and 80%, with hypercapnia (pCO_2_ 87) and acidemia (pH 6.84). He was subsequently transferred to the cardiovascular intensive care unit (CVICU) for ECLS recognizing his overall grave prognosis due to the traumatic injuries.

Venovenous ECLS was performed with the distal inflow from the inferior vena cava and proximal return in the right atrium with guidance via transesophageal echocardiography. Immediately upon initiation of ECLS, flows of 4 L/min were obtained and oxygen saturation went from 70% to 100%. Objective clinical assessment of the patient's perfusion was markedly improved. However, approximately three hours after starting ECLS, flow rates began to decrease to as low as 0.75 L/min. Hypercapnia, hypoxemia, and increasing abdominal distension with IAP measured at 45 mmHg suggested a diagnosis of overt ACS compromising ECLS function.

As the patient was too unstable for transport, an urgent bedside decompressive laparotomy was performed under intravenous anesthesia. Paracentesis was considered, but as the patient was felt to be pre-arrest with suspected ongoing dysoxia, urgent laparotomy as the ultimate therapy was selected as the most definitive and certain. A standard midline incision was carried down to the linea-alba fascia to open the peritoneum. Upon decompression, the viscera extruded spontaneously from the abdominal cavity accompanied by liters of clear serous fluid. There was a remarkable instantaneous improvement in both the ECLS flow rates (over 4 L/min) as well as the hypercapnia and hypoxemia. An open abdomen technique was used to provide temporary abdominal coverage using a local modification of the Barker VAC-PAC [6].

The patient remained in the CVICU on ECLS support for the subsequent 2 days and was slowly weaned from the ECLS circuit. Decannulation was uncomplicated and the patient's oxygen saturation following was satisfactory. Unfortunately, a CT scan of the head, electroencephalography, and clinical assessment revealed severe encephalopathy from the original anoxic insult. Upon review with the family, the decision was made to withdraw care due to a dismal prognosis.

## 3. Discussion

Despite consensus recommendations that IAP be liberally monitored in critically ill patients due to the high incidence of IAH [1], and despite the relatively frequent need for ECLS in ARDS, only 22 cases in the literature can be found where IAH was identified and linked to decreased ECLS flow [7–16]. The majority of these, 19 (86%), were identified in the pediatric population [8, 11–15]. Among the case reports involving adults [7, 9, 10, 16], only three (60%) patients underwent a decompressive laparotomy. The underlying circumstances and disease processes that led to the patients requiring ECLS were varied. Only one adult case was similar to our report in that the initial insult was caused by a motor vehicle collision. Interestingly, five pediatric cases were reported also involving submersion, respiratory failure requiring ECLS and a decompressive laparotomy for ACS [11, 15]. Similarly to all of the other published case reports where laparotomy was performed (twelve cases), our patient's hemodynamics and biochemical parameters improved initially following decompression [7, 9–11, 15, 16]. This case is unique because of the combination of rare circumstances (adult trauma involving submersion, hypothermia, and ARDS requiring ECLS, followed by ACS requiring laparotomy). Unfortunately, our patient died as a result of his anoxic brain injury. Only 5 patients among 22 published cases survived (3 children and 2 young adults) [7–9, 11, 12].

The patient's bedside laparotomy was performed outside of a traditional operating room, in the critical care unit. While this caused great consternation for the operating room staff who were asked to attend the CVICU, there is limited but well established precedent for this practice. The most urgent and widely agreed upon indication for this practice is for the decompression of overt ACS in patients who are too profoundly unstable to tolerate transport to a formal operating room [17–20]. Mayberry expressed the opinion that all those who are involved in the care of critically ill patients should be aware of these concepts and protocols developed for “*surgery outside the operating room*” prior to their need in emergent situations [19]. While the presence of an anesthesiologist is highly desirable, an intensivist experienced in intravenous anesthetic techniques may often suffice [19] (i.e., similar to intensivist sedation for percutaneous tracheostomies). A critical factor in considering bedside laparotomy is the accurate assessment of whether there is a great risk of hemorrhage [19]. De Waele reported that upon critical analysis, hemorrhagic shock was the cause of postdecompressive death in up to half of patients decompressed in certain series [21, 22]. Thus, surgeons must balance the risks of transport versus the perceived need for the improved sterility, lighting, and potentially more equipment and supplies. An expedient review of our patient's prior CT imaging reaffirmed a paucity of blood in the peritoneal cavity.

This case illustrates several salient points. The first is the critical importance of maintaining vigilance in the care of patients on ECLS. In a setting without obvious abdominal injury, an abdominal etiology resulted in a rapid precarious decline in ECLS flows. It is critical for care providers to consider more cryptic etiologies of rapid patient demise. ECLS can be an extraordinary life-saving measure but it requires extraordinary expertise and attention to remain watchful for potential challenges and complications. The additional logistical issue surrounds the recognition of the occasional necessity to perform decompressive laparotomies outside formal operating rooms. Clearly every institution that cares for critically ill patients should develop protocols to support emergent surgery outside the operating room.
